# Serum IL-6: a candidate biomarker for intracranial pressure elevation following isolated traumatic brain injury

**DOI:** 10.1186/1742-2094-7-19

**Published:** 2010-03-11

**Authors:** Georgene W Hergenroeder, Anthony N Moore, J Philip McCoy, Leigh Samsel, Norman H Ward, Guy L Clifton, Pramod K Dash

**Affiliations:** 1 The Department of Neurosurgery, The University of Texas Medical School, Houston, Texas, USA; 2 The Vivian L Smith Center for Neurologic Research, The University of Texas Medical School, Houston, Texas, USA; 3 The Department of Neurobiology and Anatomy, The University of Texas Medical School, Houston, Texas, USA; 4 National Heart Lung and Blood Institute, National Institutes of Health, Bethesda, MD, USA

## Abstract

**Background:**

Increased intracranial pressure (ICP) is a serious, life-threatening, secondary event following traumatic brain injury (TBI). In many cases, ICP rises in a delayed fashion, reaching a maximal level 48-96 hours after the initial insult. While pressure catheters can be implanted to monitor ICP, there is no clinically proven method for determining a patient's risk for developing this pathology.

**Methods:**

In the present study, we employed antibody array and Luminex-based screening methods to interrogate the levels of inflammatory cytokines in the serum of healthy volunteers and in severe TBI patients (GCS≤8) with or without incidence of elevated intracranial pressure (ICP). De-identified samples and ELISAs were used to confirm the sensitivity and specificity of IL-6 as a prognostic marker of elevated ICP in both isolated TBI patients, and polytrauma patients with TBI.

**Results:**

Consistent with previous reports, we observed sustained increases in IL-6 levels in TBI patients irrespective of their ICP status. However, the group of patients who subsequently experienced ICP ≥ 25 mm Hg had significantly higher IL-6 levels within the first 17 hours of injury as compared to the patients whose ICP remained ≤20 mm Hg. When blinded samples (n = 22) were assessed, a serum IL-6 cut-off of <5 pg/ml correctly identified 100% of all the healthy volunteers, a cut-off of >128 pg/ml correctly identified 85% of isolated TBI patients who subsequently developed elevated ICP, and values between these cut-off values correctly identified 75% of all patients whose ICP remained ≤20 mm Hg throughout the study period. In contrast, the marker had no prognostic value in predicting elevated ICP in polytrauma patients with TBI. When the levels of serum IL-6 were assessed in patients with orthopedic injury (n = 7) in the absence of TBI, a significant increase was found in these patients compared to healthy volunteers, albeit lower than that observed in TBI patients.

**Conclusions:**

Our results suggest that serum IL-6 can be used for the differential diagnosis of elevated ICP in isolated TBI.

## Background

Traumatic brain injury (TBI) is a leading cause of morbidity and mortality among civilian and military populations. The initial injury sets in motion a number of cellular and molecular events leading to the development of secondary processes that profoundly influence outcome. One of the major secondary pathologies is elevated intracranial pressure (ICP). If not maintained below 20 mm Hg, elevated ICP can cause poor cerebral perfusion, brain herniation, and death. In many cases, ICP rises in a delayed manner, reaching its peak level between days 3-5 post-TBI [1]. Although no prophylactic treatment is currently available to prevent elevated ICP, neurointensivists and neurosurgeons often use sedatives, mannitol, hypertonic saline, cerebrospinal fluid drainage, decompressive craniectomy, and/or barbiturate-induced coma to manage this condition. Therefore, the identification of prognostic biomarkers that can predict which patients are at risk of developing high ICP would help in managing severe TBI patients.

Previous studies have implicated inflammatory processes in ICP elevation [2,3]. Both pro- and anti-inflammatory cytokines have been reported to change as a result of TBI and their combined action is thought to determine the overall degree of inflammation [4]. Interleukin-6 (IL-6) is a 20-30 kDa cytokine with pleiotropic properties that has been shown to be a biomarker associated with various disease states. For example, high serum IL-6 correlates with coronary instability and carotid plaques, has been shown to be a prognostic marker for septic shock, and is an indicator of outcome in severe intra-abdominal sepsis [5-7]. In TBI, a relationship has been reported between the transcranial IL-6 gradient (venous-arterial) at the time of admission and survivability [8]. In addition, a recent multiplex analysis of putative serum biomarkers identified IL-6 as a marker of inflicted pediatric TBI [9]. Although these studies support the premise that inflammation-related proteins such as IL-6 are elevated following TBI, it has not been examined if the magnitude or duration of induction correlates with the development of ICP.

In the present study, we utilized two screening methods, antibody arrays and multiplexing, to evaluate the levels of interleukin family members in the serum of healthy volunteers and in severe TBI patients (GCS≤8) with and without incidence of ICP. Consistent with a number of previous reports, we observed elevated IL-6 levels in TBI patients as compared to healthy volunteers. Interestingly, the group of TBI patients who subsequently developed ICP ≥ 25 mm Hg had significantly higher serum IL-6 levels within the first 17 hours of injury as compared to the patients whose ICP remained ≤20 mm Hg during their hospital stay. However, in polytrauma patients with TBI, serum IL-6 levels were unable to differentiate between the two groups. IL-6 levels in patients with orthopedic injury were found to be higher than those recorded in healthy volunteers, but lower than seen in the TBI patients. Taken together, our results suggest that for patients with isolated TBI, serum IL-6 is a good prognostic biomarker for predicting subsequent ICP elevations.

## Methods

### Recruitment and sample collection

All protocols regarding the use of human subjects were reviewed and approved by the University of Texas Committee for the Protection of Human Subjects, and were in compliance with the Helsinki Declaration. Blood samples were obtained specifically for the purpose of this study and were coded to protect confidentiality. Non-trauma volunteers were consented and enrolled by the University of Texas Clinical Research Unit at Memorial Hermann Hospital (Houston, TX). To be included in the study, orthopedic injury patients admitted to the Memorial Hermann Hospital Emergency Department had to have a radiographically confirmed fracture, no head trauma, no other known inflammatory process or infection, no history of neurological or psychiatric disorders, and no alcohol or drug dependency. Orthopedic injury subjects were consented and a one-time blood draw within 24 hr of the injury was taken. TBI patients (14-65 years old) admitted to the adult neurotrauma intensive care unit (NTICU) of Memorial Hermann Hospital who had Glasgow Coma Scale (GCS) scores ≤8 and planned placement of an ICP monitor were recruited, and consented (through their next-of-kin) for participation in this study. ICP measurements were measured continuously and recorded hourly from the time of monitor placement. Elevated ICP was defined as a ≥25 mm Hg measurement for at least 5 minutes that occurred either twice in a 24 hr period, or on two consecutive days. No change in ICP was defined as an ICP level of ≤20 mmHg throughout the 5 day study period. Patients whose ICP fell between 20-25 mmHg on any of the sampling days were not included in the present study in order to provide a clear separation between the two injury groups. An initial blood draw was obtained at the earliest possible time after admission, patient stabilization, and informed consent was obtained. To minimize interference with clinical management, subsequent blood samples were drawn after morning rounds were completed (i.e. every 24 hours for first five days of hospital admission). Relevant medical data was recorded and coded to match the extracted blood sample. Serum was isolated by centrifugation at 4°C using serum separator tubes (Becton Dickinson, Franklin Lakes, NJ) as described by the vendor. Aliquots were prepared and frozen at -80°C until needed.

### Patient management

All patients admitted to the NTICU of Memorial Hermann Hospital received standard care based on the types and severities of their injuries. Patients were managed to avoid hypoxia and hypotension, and to attempt to maintain adequate cerebral blood flow (*e.g*., systolic pressure above 90 mm Hg and a cerebral perfusion pressure (CPP) of at least 60 mm Hg.). Head computed tomography (CT) scans were performed on presentation to the Emergency Department and repeated within approximately 6 hours. Subsequent CTs were performed based on the patient's neurological status. Intracranial pressure monitors were placed in those with a GCS ≤ 8, or those with significant findings on head CT who in the opinion of the attending neurosurgeon required intracranial pressure monitoring. Any patient with an operative lesion was transferred to the operating room emergently. The decision to remove a bone flap was made by the attending neurosurgeon at his discretion. Medications to prevent seizures were given prophylactically for the first 7 days of hospitalization. The patient's temperature was controlled to prevent the patient from becoming hyperthermic. Patient's intake and output were monitored to assure that the patient received adequate fluid volume. Sedation and analgesics were used in all severe TBI patients. Drainage of CSF, mannitol and/or hypertonic saline was used, as required, to lower intracranial pressure. Refractory elevated ICP typically went through the treatment continuum of sedation, mannitol, CSF drainage, paralytics, barbiturate coma, and decompressive craniectomy. With the exception of those treatments initiated to control intracranial pressure (e.g. mannitol, cranieotomy, etc.), the care did not differ between the two study groups.

### Patient assessments and classifications

Acute Physiology and Chronic Health Evaluation II (APACHE II), GCS and Injury Severity Score (ISS). APACHE II is designed to measure the severity of disease for newly admitted ICU patients [10]. The worst value for each component of the scale in the first 24 hours of ICU admission was collected. GCS is a neurological assessment routinely used in clinical care and neurosurgical research [11]. All injuries were classified according to body region and given an abbreviated injury score (AIS, The Abbreviated Injury Scale, 1990 Revision, Update 98) from 1 to 6 according to standardized definitions (a score of 6 indicates death). The worst three AIS scores in different body regions were squared and summed to calculate the ISS. The ISS correlates linearly with mortality, morbidity, length of hospital stay, and other measures of injury severity [12]. Polytrauma was defined as a head injury along with an AIS score ≥3 in a body region other than the head, neck or face.

### Human cytokine array

The levels of inflammation-related factors were assessed using the Cytokine Array 2000 series (Ray Biotech, Norcross, GA) as described by the vendor. Serum samples (n = 14/group) collected within the first 24 hr of injury were chosen from TBI patients whose ICP remained ≤20 mm Hg throughout the study period, and from TBI patients whose maximum recorded ICP ≥ 25 mm Hg. Samples were divided (n = 7/time point) based on their collecting time into either 10 hr (± 4 hr) or 20 hr (± 4 hr) groups, then pooled. Pooled samples from age-, race- and gender-matched healthy volunteers served as baseline controls. The membranes were blocked against non-specific binding for 2 hr using the 1× blocking buffer provided in the assay kit. Pooled serum samples were diluted 10-fold in 1× blocking buffer and incubated on individual membranes overnight at 4°C with gentle shaking. Following washing, biotinylated antibodies were added for 2 hr. The binding of the biotinylated antibodies was detected using a horseradish peroxidase-streptavidin complex and a chemilumenescent substrate. Signals were captured on BioMax light film (Perkin-Elmer, Waltham, Massachusetts) and analyzed using *Image J *densitometric software. All groups were assessed simultaneously so that they were represented on the same film. Multiple exposures were generated to ensure the linearity of the signals. The cumulative optical densities of the resultant spots on each membrane were summed and compared across membranes for normalization purposes. Antibodies against each analyte were represented in duplicate spots on each membrane, the optical densities of which were averaged. Each pooled sample was tested on two independent arrays.

### Luminex

The levels of IL-1α, IL-1β, IL-2, IL-4, IL-5, IL-6, IL-10, IL-17, and IL-1ra were determined using Luminex and kits obtained from R&D Systems (Minneapolis, MN) and the procedures were conducted as recommended by the vendor. Briefly, a standard curve for each of the target proteins was prepared by serial dilution in assay buffer. Serum samples were diluted 1:4. Standards and samples were incubated in a 96-well filter-bottom plate with target-specific microparticles for 2 hr at room temperature with shaking. Following the incubation, microparticles were washed 3 times using the wash buffer provided in the kit and a vacuum manifold. Biotinylated antibodies were added and incubated for 1 hr, and detected using a streptavidin-phycoerythrin conjugate. Following extensive washing, samples were analyzed on a Luminex plate reader. All samples were analyzed in duplicate and the concentrations calculated by comparison to the appropriate standard curve.

### Enzyme-linked immunosorbent assays (ELISAs)

To test the predictive power of changes in serum IL-6 proteins, sandwich ELISAs were performed using samples collected during the first 24 hr of injury. Samples from healthy volunteers were used as controls. Samples were coded so that the experimenter was unaware of the group designation during the procedure. A standard curve was prepared by serial dilution of purified recombinant protein supplied with the assay (R&D systems, Minneapolis, MN). Standards and serum samples (in triplicate) were added to a 96-well plate containing immobilized antibodies specific to IL-6. Samples were incubated at room temperature for 2 hours, with gentle shaking. Following extensive washing, bound IL-6 was detected using an HRP-conjugated polyclonal antibody against the target protein. The detection antibody was incubated for 2 hr at room temperature, and detected using 2,2'-Azino-bis-(3-ethylbenzthiazoline-6-sulfonic acid) and 3% H_2_O_2_. The reaction was terminated by the addition of sulfuric acid provided in the kit. The resultant optical density was detected using a microplate reader at 450 nm with a wavelength correction reading at 540 nm. The concentrations of the unknowns were calculated from the standard curve using *SigmaPlot *(Systat Software, Inc, San Jose CA).

### Statistical analysis

A power analysis based on approximated magnitudes of change and technique reproducibility was performed *a priori *to estimate the group sizes expected to provide statistically significant results. All data was assessed using a Kolmogorov-Smirnov test to determine normal distribution. Time course data was compared using a two-way ANOVA. Data for two-group or multiple group comparisons at a single time point were analyzed using a Students *t-test *or one-way ANOVA, respectively. Any data that did not have a normal distribution was assessed using non-parametric measures (e.g. ANOVA on Ranks). Data are presented as the mean ± SEM unless otherwise indicated, and the median value and data range are provided where appropriate.

## Results

### Interleukin family member expression in the serum of TBI patients with/without elevated ICP

To determine if the serum levels of interleukin family members can be used to differentiate TBI patients based on whether or not they will have a subsequent elevation in ICP, two screening approaches were utilized: 1) antibody arrays using pooled serum samples from healthy volunteers and TBI patients with/without incidence of ICP ≥ 25 mm Hg, and 2) Luminex analysis of individual serum samples from independent patients belonging to the same groups.

#### Experiment 1: Antibody array

**Figure 1:**
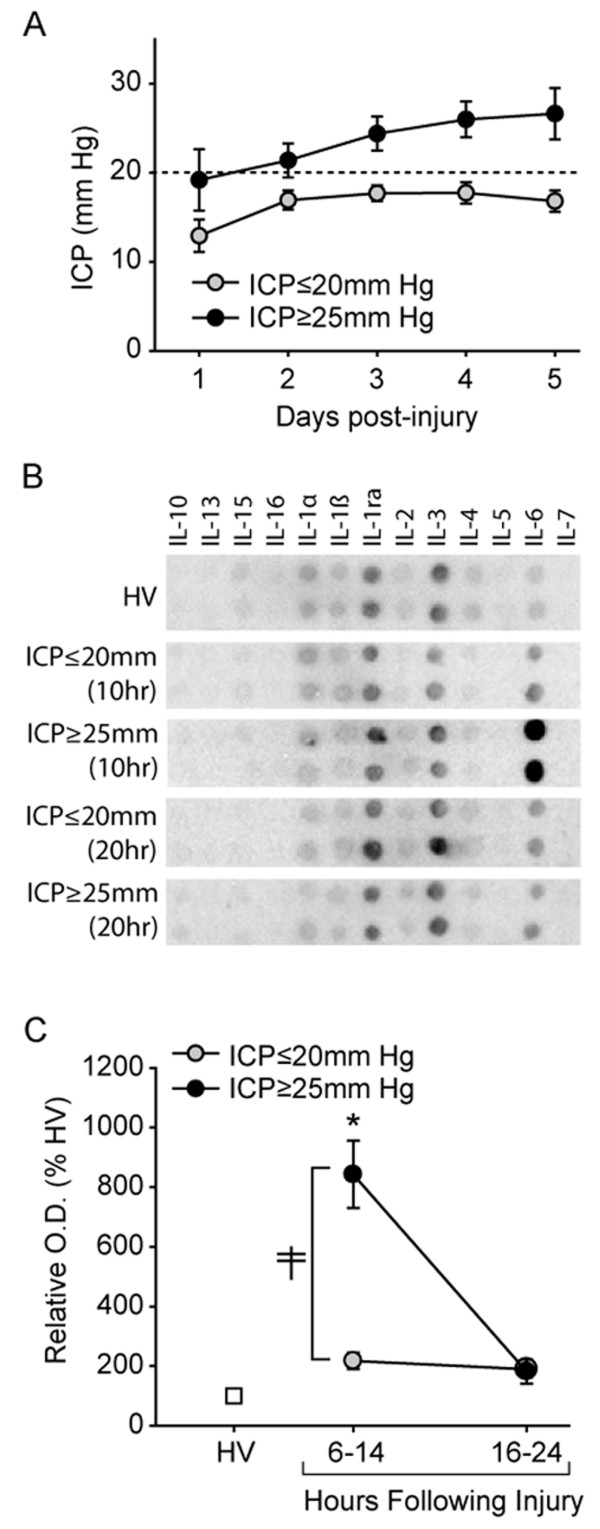
**Identification of serum IL-6 as a marker of elevated ICP by antibody arrays**. **A**. Time course of ICP in patients classified as having elevated ICP (ICP ≥ 25 mm Hg) versus that recorded in patients whose ICP remained below 20 mm Hg for the duration of the sampling period. **B**. Representative picture of a Ray Biosciences Cytokine array showing the immunoreactivity of interleukin family members in pooled samples (n = 7/pool) from healthy volunteers, TBI patients with elevated ICP, and TBI patients whose ICP remained below 20 mm Hg for the duration of the 5 day sampling period. **C**. Summary data (presented as % healthy volunteer(HV)) showing that the relative signal of IL-6 is significantly increased in patients with ICP ≥ 25 mm Hg compared to patients whose ICP remained below 20 mm Hg throughout the study period. Data is presented as mean ± SEM. ‡, significant difference by two-way ANOVA.

Using the recorded ICP values for the TBI patients enrolled in this study, patients were divided into either elevated ICP (ICP ≥ 25 mm Hg) or no-ICP groups (ICP ≤ 20 mm Hg). Age-, race- and gender-matched healthy volunteers were selected and used as controls. The demographics of the patients used in this study are listed in Table 1. Figure 1A shows that when the maximum recorded ICP levels each day were plotted as a function of time, the elevated ICP group had a delayed increase in ICP, reaching a maximum between days 3 and 5 post-injury. By comparison, the patients without incidence of elevated ICP had pressure values that remained below the treatment level of 20 mm Hg throughout the study period. Although it appears that the initial ICP values in the ICP ≥ 25 mm Hg group are higher than those detected in the ICP ≤ 20 mm Hg group, correlation analysis revealed that initial ICP is not a strong predictor of subsequent ICP elevations. When the ICP values were analyzed to determine if there was a correlation between initial ICP value (day 1) and those recorded on days 3-5 (the time points at which ICP ≥ 25 mm Hg were observed), correlation coefficients of 0.596 (day 3), 0.241 (day 4), and 0.173 (day 5) were calculated.

**Table 1:** Demographic and clinical information for subjects used for pooled samples for RayBioscience assays.

	Healthy Volunteers (n = 14)	ICP ≤20 mm Hg (n = 14)	ICP ≥25 mm Hg (n = 14)
**Age range**	22-39 years	15-48 years	14-56 years

**Age (average)**	26.2 ± 4.7 years	24.4 ± 9.2 years	28.1 ± 14.8 years

**Gender**	11 male	12 male	12 male
	3 female	2 female	2 female

**Race**	10 Caucasians	11 Caucasians	12 Caucasians
	3 African-Americans	3 African-Americans	1 African-American
	1 Asian		1 Asian

**Ethnicity**	11 non-hispanics	9 non-hispanics	6 non-hispanics
	3 non-hispanics	5 hispanics	8 hispanics


**Apache II score**	N/A	18.8 ± 6.0	20.2 ± 4.1

**ISS**	N/A	27.9 ± 6.4	23.0 ± 8.4

Data is presented as mean ± SEM.

As the principle aim of the current study was to determine if the initial levels of interleukin family members could be used to identify patients at risk for developing elevated ICP, serum samples from each of the groups were further subdivided based on their collection time. Pools of samples were prepared corresponding to either 10 ± 4 hr or 20 ± 4 hr post-injury (n = 7/group), and applied to a cytokine array as directed by the vendor (Ray Biosciences). Figure 1B shows representative images of the resultant blots indicating the relative immunoreactivities for IL-10, IL-13, IL-15, IL-16, IL-1α, IL-1β, Il-1ra, IL-2, IL-3, IL-4, Il-5, IL-6 and IL-7. Quantification of the optical densities for these cytokines revealed that while the serum levels of IL-6 were dramatically elevated in both TBI groups relative to that observed in the healthy volunteers, the magnitude of increase seen in the TBI patients with ICP ≥ 25 mm Hg was significantly higher than that seen in the TBI patients whose ICP remained ≤20 mm Hg throughout the study period (relative immunoreactivity: healthy volunteers = 100.0 ± 16.96%, ICP ≤ 20 mm Hg = 217.5 ± 26.95%, ICP ≥ 25 mm Hg = 842.7 ± 112.96%; significant interaction by two-way ANOVA F_(1,8) _= 12.835, P = 0.007) (Figure 1C). No significant change was detected in any of the other cytokines interrogated.

#### Experiment 2: Luminex

**Figure 2:**
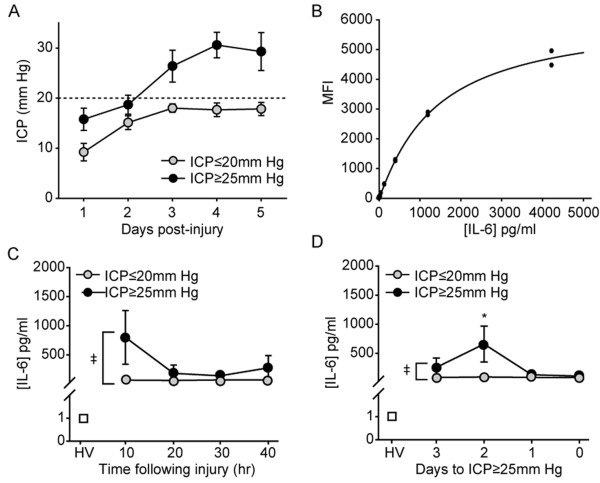
**Early increase in serum IL-6 can be used to stratify TBI patients based on risk for developing elevated ICP**. **A**. Time course of ICP in patients classified as having elevated ICP (ICP > 25 mm Hg) versus that recorded in patients whose ICP remained below 20 mm Hg for the duration of the sampling period. **B**. Standard curve showing the relationship between increasing IL-6 concentrations and the mean fluorescent intensity (MFI) detected by Luminex analysis. **C**. Summary data showing the levels of IL-6 detected in the serum of healthy volunteers (HV), from TBI patients with elevated ICP (ICP > 25 mm Hg), and from patients whose ICP remained below 20 mm Hg throughout the study period. **D**. Luminex data organized based on time of sample withdrawal relative to the subsequent increase in ICP. Day 0 represents the time point at which the ICP was recorded to surpass 25 mm Hg. Each patient with an elevation in ICP was paired with a patient whose ICP remained ≤20 mm Hg throughout the study period. Data is presented as mean ± SEM. ‡, significant difference by two-way ANOVA.

Since the above analysis was performed using pooled samples, it raised the possibility that a large increase in IL-6 levels within a few of the patients would have been sufficient to increase the level of this protein in the entire pooled sample. To address this concern, Luminex analysis was performed using individual serum samples from independent study subjects. The demographics of the subjects used in this study are presented in Table 2. Figure 2A shows that these patients, similar to those chosen for the antibody array screen, had ICP levels that either remained ≤20 mm Hg throughout the study period, or had a delayed increase in ICP that necessitated intervention. As in Experiment 1, the ICP values recorded for these patients on day1 was found to not be a strong predictor of the maximum ICP values recorded on day3 (correlation coefficient = 0.626), day4 (correlation coefficient = 0.353), or day5 (correlation coefficient = 0.152).

**Table 2:** Demographic and clinical information for subjects used for Luminex analysis. Data is presented as mean ± SEM.

	Healthy Volunteers (n = 10)	ICP ≤20 mm Hg (n = 9)	ICP ≥25 mm Hg (n = 10)
**Age range**	20-39 years	19-51 years	14-54 years

**Age (average)**	26.5 ± 5.5 years	29.5 ± 12.1 years	29.3 ± 11.2 years

**Gender**	8 male	6 male	9 male
	2 female	3 female	1 female

**Race**	8 Caucasians	8 Caucasians	9 Caucasians
	1 African-Americans	1 African-Americans	1 African-American
	1 Asian		

**Ethnicity**	5 non-hispanics	6 non-hispanics	6 non-hispanics
	5 non-hispanics	3 hispanics	4 hispanics

**Apache II score**	N/A	17.6 ± 3.7	21.2 ± 3.5

**ISS**	N/A	23.6 ± 5.4	19.8 ± 3.6

**Therapy intensity level**			
*first 24 hr*	N/A	4.0 ± 1.8	5.1 ± 4.1
*72-120 hr*	N/A	3.2 ± 1.8	5.2 ± 4.0

**Mortality at time of discharge**	N/A	0/9 (0%)	2/10 (20%)

**Mechanism of injury**	N/A		
*motorcycle collision*		3/9 (33%)	3/10 (30%)
*motor vehicle accident*		5/9 (55%)^a^	5/10 (50%)
*fall*		0/9 (0%)	2/10 (20%)^b^
*assault*		1/9 (11%)	0/10 (0%)

**Initial CT results^c^**	N/A		
*skull fracture*		5/9 (56%)	5/10 (50%)
*subarachnoid hemorrhage*		6/9 (67%)	7/10 (70%)
*epidural hematoma*		3/9 (33%)	1/10 (10%)
*subdural hematoma*		3/9 (33%)	8/10 (80%)
*intraventricular hemorrhage*		4/9 (44%)	3/10 (33%)
*contusion*		7/9 (78%)	8/10 (80%)
*midline shift*		3/9 (33%)	3/10 (30%)
*edema*		5/9 (56%)	3/10 (30%)
*diffuse axonal injury*		0/9 (0%)	1/10 (10%)

^a^, one subject was assaulted after motor vehicle accident

^b^, one subject fell off a moving vehicle

^c^, some patients may present with more than one pathology.

Luminex analysis was performed using analyte kits available for multiplexing from R&D Systems (Minneapolis, MN). The interleukin family members interrogated were IL-10, IL-17, IL-1α, IL-1β, IL-1ra, IL-2, IL-4, IL-5, and IL-6. Standard curves for each of the analytes were generated using purified proteins supplied by the vendor, and used to calculate the concentration of the target protein in each of the individual samples. A representative standard curve generated for IL-6 is shown in Figure 2B. Similar to that observed using the antibody array, when the levels of serum IL-6 were compared between the healthy volunteers and TBI patients, a dramatic increase in the level of this protein was observed as a result of injury (Figure 2C). Of importance is the observation that the initial levels of IL-6 recorded in TBI patients that would subsequently develop elevated ICP were significantly higher than that observed in TBI patients whose ICP remained below 20 mm Hg (group main effect by two-way ANOVA F_(1,3) _= 6.541, P = 0.016). When organized according to time from ICP (the day ICP was first recorded to exceed 25 mm Hg was designated as day 0), the significant increase in IL-6 levels was observed to occur, on average, two days prior to the recorded time of ICP elevation (group main effect by two-way ANOVA F_(1,3) _= 4.040, P = 0.049) (Figure 2D). All other cytokines, with the exception of IL-1ra, were below the detection limit of the Luminex assay. Serum IL-1ra (data not shown), although elevated as a result of TBI, was not significantly different between the ICP groups.

### Prognostic accuracy of IL-6 for predicting ICP in TBI patients

Based on the results of the Luminex screening, cut-off values of ≥128 pg/ml (ICP ≥ 25 mm Hg), 5.1-127.9 pg/ml (for ICP ≤ 20 mm Hg), and ≤5 pg/ml (for healthy volunteers) serum IL-6 were identified as being able to stratify the patient groups. To test the accuracy of these cut-off values, ELISAs were performed using samples that were blinded as to their group designations. Prior to initiating these studies, randomly chosen serum samples used for Luminex analysis were re-probed using an IL-6 ELISA to determine the consistency of the measurements. Table 3 shows the recorded values for each of the samples. The table shows that 7 out of 8 (87.5%) samples identified as having serum IL-6 values ≥128 pg/ml by Luminex also giving values ≥128 pg/ml by ELISA. Similarly, 75% of samples with serum IL-6 concentrations between 5.1-127.9 pg/ml were found to be in this range by ELISA.

**Table 3:** Comparison of IL-6 concentrations detected in 20 randomly chosen serum samples detected using Luminex and conventional ELISA analyses.

Sample	Luminex	ELISA	Sample	Luminex	ELISA
1	69.8 pg/ml	76.3 pg/ml	11	130.8 pg/ml	153.1 pg/ml

2	62.5 pg/ml	69.4 pg/ml	12	902.1 pg/ml	54.7 pg/ml

3	883.5 pg/ml	>600 pg/ml	13	268.2 pg/ml	322.2 pg/ml

4	116.2 pg/ml	148.6 pg/ml	14	47.4 pg/ml	57.5 pg/ml

5	110.1 pg/ml	179.7 pg/ml	15	28.9 pg/ml	61.7 pg/ml

6	154.6 pg/ml	172.5 pg/ml	16	204.4 pg/ml	257.0 pg/ml

7	43.6 pg/ml	63.5 pg/ml	17	57.2 pg/ml	93.4 pg/ml

8	83.5 pg/ml	124.2 pg/ml	18	59.7 pg/ml	89.7 pg/ml

9	883.5 pg/ml	>600 pg/ml	19	160.1 pg/ml	204.5 pg/ml

10	28.1 pg/ml	43.5 pg/ml	20	30.7 pg/ml	36.9 pg/ml

De-identified samples from healthy volunteers and TBI patients, whose first sample draw occurred during the first 17 hr of injury, were analyzed for IL-6 levels using ELISA. This time point was chosen as it represented the latest time at which IL-6 serum concentrations were found to be consistently elevated in the ICP ≥ 25 mm Hg group by the antibody array and Luminex analyses. Predetermined cut-off values, based on the Luminex analysis, of ≤5 pg/ml for healthy volunteers, 5-127.9 pg/ml for ICP ≤ 20 mm Hg and ≥128 pg/ml for ICP ≥ 25 mm Hg, were used to classify the de-identified samples. Table 4 shows the measured concentrations of IL-6 in these samples, along with the predicted and actual group designations. The results show that serum IL-6 concentrations could be used to correctly classify 7 of 7 healthy volunteers, and 15 of 15 injured patients. Of the injured patients, 6 of 8 were correctly identified as belonging to the ICP ≤ 20 mm Hg group, and 6 of 7 correctly identified as having a subsequent ICP elevation (85.7% sensitivity, 75% specificity) at the cut-off values employed. Compilation of all the recorded IL-6 values for the ICP ≤ 20 mm Hg and ICP ≥ 25 mm Hg groups yielded a receiver operator characteristic curve with an area under the curve of 0.81, suggesting that IL-6 is a good prognostic marker for ICP in isolated brain injury.

**Table 4:** Diagnostic accuracy of IL-6 for predicting subsequent elevations in ICP.

	Maximum ICP (mm Hg)	ELISA (pg/ml IL-6)	Classification	Actual
**1**	NA	undetectable	HV	HV

**2**	NA	undetectable	HV	HV

**3**	NA	undetectable	HV	HV

**4**	NA	2.01	HV	HV

**5**	NA	undetectable	HV	HV

**6**	NA	undetectable	HV	HV

**7**	NA	undetectable	HV	HV

**8**	20	76.31	ICP ≤ 20 mm Hg	ICP ≤ 20 mm Hg

**9**	16	148.58	ICP ≥ 25 mm Hg	ICP ≤ 20 mm Hg

**10**	20	54.66	ICP ≤ 20 mm Hg	ICP ≤ 20 mm Hg

**11**	19	63.53	ICP ≤ 20 mm Hg	ICP ≤ 20 mm Hg

**12**	20	251.51	ICP ≥ 25 mm Hg	ICP ≤ 20 mm Hg

**13**	16	57.48	ICP ≤ 20 mm Hg	ICP ≤ 20 mm Hg

**14**	19	93.45	ICP ≤ 20 mm Hg	ICP ≤ 20 mm Hg

**15**	20	94.6	ICP ≤ 20 mm Hg	ICP ≤ 20 mm Hg

**16**	30	188.17	ICP ≥ 25 mm Hg	ICP ≥ 25 mm Hg

**17**	28	573.01	ICP ≥ 25 mm Hg	ICP ≥ 25 mm Hg

**18**	26	152.78	ICP ≥ 25 mm Hg	ICP ≥ 25 mm Hg

**19**	31	>600	ICP ≥ 25 mm Hg	ICP ≥ 25 mm Hg

**20**	31	172.46	ICP ≥ 25 mm Hg	ICP ≥ 25 mm Hg

**21**	38	>600	ICP ≥ 25 mm Hg	ICP ≥ 25 mm Hg

**22**	30	43.52	ICP ≤ 20 mm Hg	ICP ≥ 25 mm Hg

Blinded samples, taken within the first 16 hr of injury (or from paired healthy volunteers), were analyzed for IL-6 content. HV: healthy volunteer

**Figure 3:**
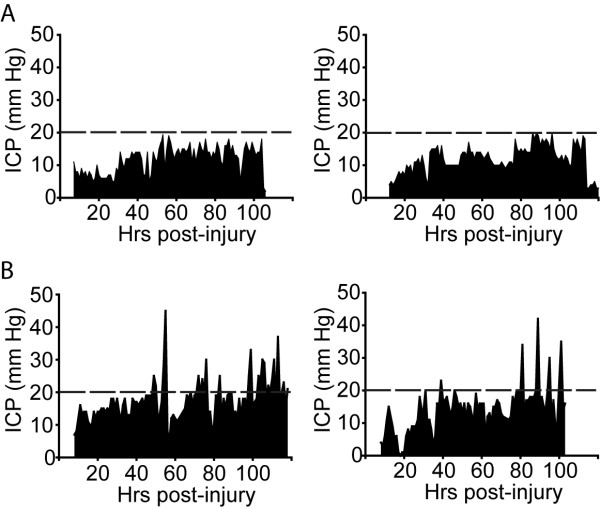
**Representative ICP profiles for TBI patients**. ICP profiles for TBI patients whose ICP **A**. remained ≤20 mm Hg throughout the 5 day study period and **B**. elevated to ≥25 mm Hg. Dotted line represents 20 mm Hg, the threshold for clinical intervention.

We next questioned if the increase in IL-6 levels observed in patients whose ICP subsequently elevated to ≥25 mm Hg could be correlated to the degree (magnitude, frequency or duration) of ICP elevation. ICP profiles from individual patients were generated using data recorded hourly by the nursing staff. Figure 3A shows representative ICP profiles from TBI patients whose ICP remained below the threshold for therapeutic intervention (20 mm Hg; dotted line). Patients with elevated ICP, by comparison, had several ICP spikes that necessitated ICP management (Figure 3B). To determine if the degree of ICP elevation correlated with initial IL-6 values, the number of spikes over 20 mm Hg, the maximum recorded ICP value, and the area under the curve (AUC) for the ICP values over 20 mm Hg were calculated for the 5 day monitoring period. Correlation analysis revealed that there was no correlation between the level of IL-6 and either the number of ICP events (correlation coefficient = -0.23), the maximum ICP value (correlation coefficient = 0.20), or the AUC (correlation coefficient = -0.24). However, since all the patients in this study received treatment if their ICP passed 20 mmHg, the ICP profiles recorded (magnitude, frequency and duration) are likely to have been influenced by the treatments being instituted for each patient.

### Prognostic accuracy of IL-6 for predicting ICP in polytrauma patients

As TBI often occurs in the presence of bodily trauma, and serum IL-6 levels have been shown to increase following other injuries, we questioned if the prognostic accuracy of this biomarker in identifying TBI patients at risk for developing elevated ICP would be compromised by polytrauma. Polytrauma was defined as a head injury along with an injury to another body region that received an AIS score ≥3. De-identified samples from these patients were analyzed for serum IL-6 content and group designations assigned using the aforementioned cut-off values. The results presented in the Table 5 show that serum IL-6 concentrations could be used to only correctly classify 6 of the 11 polytrauma patients tested (54.5%) based on their ICP status.

**Table 5:** Diagnostic accuracy of IL-6 for predicting subsequent elevations in ICP in patients with polytrauma.

	Maximum ICP (mm Hg)	IL-6 (pg/ml)	Classification	Actual
**1**	NA	undetectable	HV	HV

**2**	NA	2.06	HV	HV

**3**	NA	1.44	HV	HV

**4**	11	>600	ICP ≥ 25 mm Hg	ICP ≤ 20 mm Hg

**5**	16	140.28	ICP ≥ 25 mm Hg	ICP ≤ 20 mm Hg

**6**	20	50.01	ICP ≤ 20 mm Hg	ICP ≤ 20 mm Hg

**7**	20	518.70	ICP ≥ 25 mm Hg	ICP ≤ 20 mm Hg

**8**	12	75.00	ICP ≤ 20 mm Hg	ICP ≤ 20 mm Hg

**9**	34	85.21	ICP ≤ 20 mm Hg	ICP ≥ 25 mm Hg

**10**	32	342.89	ICP ≥ 25 mm Hg	ICP ≥ 25 mm Hg

**11**	28	189.76	ICP ≥ 25 mm Hg	ICP ≥ 25 mm Hg

**12**	38	30.91	ICP ≤ 20 mm Hg	ICP ≥ 25 mm Hg

**13**	34	236.90	ICP ≥ 25 mm Hg	ICP ≥ 25 mm Hg

**14**	26	466.18	ICP ≥ 25 mm Hg	ICP ≥ 25 mm Hg

Blinded samples taken within the first 16 hr of injury (or from paired healthy volunteers) were analyzed for IL-6 content. Serum IL-6 concentrations could be used to correctly identify only 6 of the 11 polytrauma patients tested (54%). HV: healthy volunteer.

### Increases in IL-6 levels in patients with orthopedic injury

**Figure 4:**
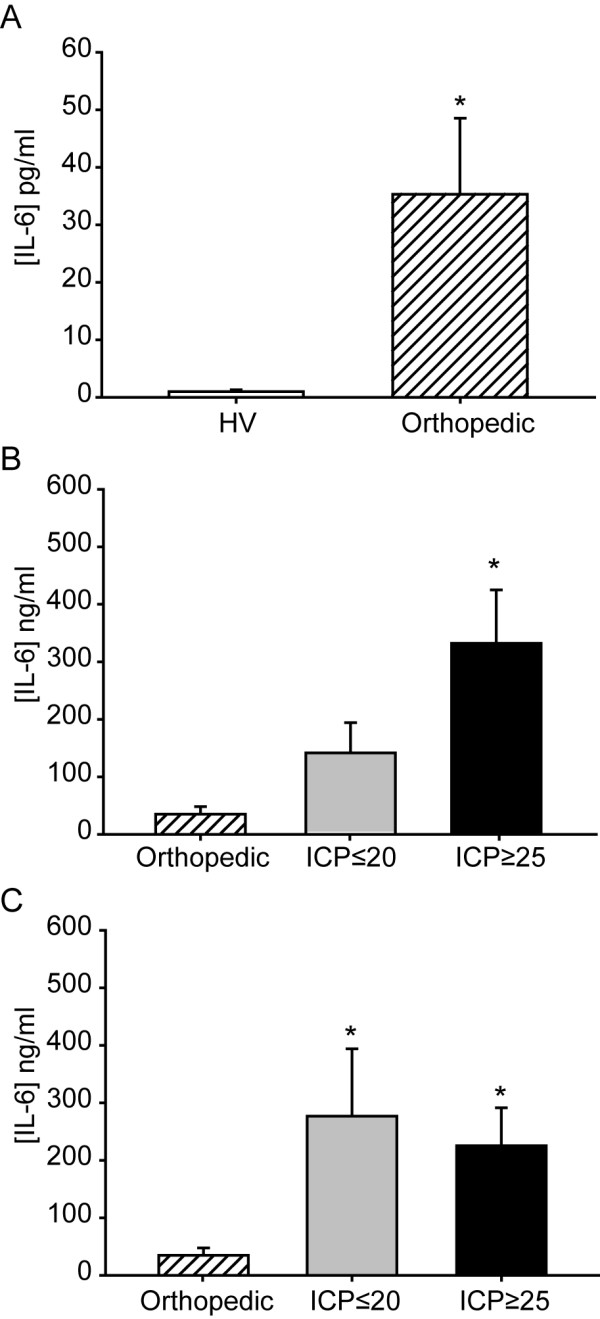
**Orthopedic injury increases serum IL-6 levels**. **A**. Summary data showing the mean serum IL-6 concentrations for healthy volunteers (HV) and orthopedic injury patients. Serum IL-6 levels were significantly lower in orthopedic injury patients compared to TBI patients with **B**. isolated brain injury and **C**. polytrauma. Data is presented as mean ± SEM. *, P < 0.05.

The loss of prognostic accuracy of serum IL-6 in polytrauma patients suggests that the presence of other bodily injuries may be altering serum IL-6 levels. To address this possibility, we measured its concentration in patients with orthopedic injury (n = 7) in the absence of a TBI diagnosis (GCS = 15). Serum samples from these patients were collected within 24 hrs of injury. Table 6 shows the type of injury and serum IL-6 concentrations recorded in these individuals. On average, serum IL-6 was found to be significantly elevated as a result of orthopedic injury as compared to healthy volunteers (healthy volunteers = 0.98 ± 0.37 pg/ml vs orthopedic injury = 35.09 ± 14.02 pg/ml, p = 0.002) (Figure 4A). Although elevated, these levels were found to be significantly lower than those detected in isolated TBI (one-way ANOVA on Ranks: H = 12.076, P = 0.002; Figure 4B) and in polytrauma patients (one-way ANOVA on Ranks: H = 8.157, P = 0.017; Figure 4C). Interestingly, while the orthopedic injury patients were found have a serum IL-6 levels that were significantly lower than both polytrauma groups (i.e. ICP ≤ 20 mm Hg and ICP ≥ 25 mm Hg), they were not different than that observed in the isolated TBI patients whose ICP remained lower than 20 mm Hg (Figure 4B and 4C). This suggests a general increase in serum IL-6 levels as a result of polytrauma.

**Table 6:** Serum IL-6 levels in orthopedic injury patients without diagnosis of brain injury.

Patient	Injury	ELISA (pg/ml IL-6)
**1**	Right metacarpel fracture	14.68

**2**	Open tibia/fibia fracture, fractures of lumbar vertebrae and coccyx	100.89

**3**	Crush injury to right leg	24.63

**4**	Communited fracture of right femur midshaft	53.88

**5**	Bimalleolar fracture of ankle	1.71

**6**	Crushed foot, closed tibia and fibia fracture	31.93

**7**	Trimalleolar fracture of right ankle	7.21

Samples were taken within the first 24 hr of injury.

## Discussion

In the present study, we employed two independent cytokine screening platforms to identify serum IL-6 as a prognostic biomarker for ICP elevation following TBI. To the best of our knowledge, this is the first report to indicate that serum IL-6 levels can be used as a prognostic biomarker for elevated ICP in patients with isolated TBI. Although we observed a change in serum IL-6 levels in TBI patients, the present study does not indicate if the increased IL-6 we measured originated in the injured brain, or was the result of a peripheral response to the injury. Previous studies have shown that injury to the brain can cause both localized and systemic inflammatory reactions [13-15]. In the brain, neurons, astrocytes and microglia have all been shown to express IL-6 [16-19], and it has been argued that this production is the main source of IL-6 in the serum following TBI. For example, arterial and jugular differences in IL-6 concentration have been observed in TBI patients [8,20]. Furthermore, IL-6 is the cytokine that is present in the highest concentration in human CSF after TBI [21], reaching peak concentrations of up to 30,000 pg/ml [22]. By comparison, peak serum concentrations of IL-6 have been reported to only reach 1090 pg/ml during the same time period [23]. Taken together, these studies suggest that the increase in serum IL-6 levels we observed may have originated in the injured brain, rather than the result of a peripheral response to the injury.

A number of studies have reported that increased IL-6 levels in the CSF correlate with favorable outcome after brain injury [8,24-26]. For example, an *in vivo *microdialysis study has shown that increased parenchymal IL-6 levels following TBI correlates with survival and improved Galsgow Outcome Score (GOS) [26]. In addition, Chiaretti et al., [27] reported that early increases in CSF IL-6 levels are associated with improved neurologic outcome in children with severe TBI. In contrast to these influences of IL-6 in the CSF, elevated serum IL-6 has been shown to correlate with multi-organ failure and death following TBI [28,29], and is associated with poor neurological outcome following hemorrhagic stroke [30]. Serum levels of IL-6 ranging from 663.3 pg/mL to 1060.9 pg/mL have been documented to occur following brain death [31]. In non-CNS diseases, high serum IL-6 levels have been correlated to poor survival of breast cancer [32], are thought to be proatherogenic [33], and are strong predictors of mortality in patients with end-stage renal disease [34]. The reason for these apparent differential effects of IL-6 is not known, but may be dependent on the affected cell types. For example, IL-6 has been reported to reduce glutamate- and NMDA-induced neuronal death [35,36], and its loss is associated with increased oxidative stress and neurodegeneration after injury [37]. In contrast, transgenic mice overexpressing IL-6 in glial cells show ataxia, seizures and extensive neurodegeneration [38]. Although the relationship between high serum IL-6 and the development of ICP is not clear at present, our findings are consistent with previous observations that high serum IL-6 levels are often associated with poor outcome. Future basic and clinical studies using IL-6 receptor blockers such as *Tocilizumab *(an anti-IL6R used to treat rheumatoid arthritis) are needed to establish a role for IL-6 in the progression of this secondary pathology.

In polytrauma patients, IL-6 can also be released into the circulation as a result of injury to other organs. Many cell types including monocytes, macrophages, fibroblast, keratinocytes, smooth muscle cells, T cells, and B cells have all been shown to increase IL-6 production in response to injury [39-43]. This extracranial production would be expected to further elevate serum IL-6 and exacerbate outcome. Consistent with this, Hensler et al., [44] reported that the serum concentrations of a number of cytokines, including IL-6, in patients with multiple injuries with TBI is higher than that observed in isolated TBI. Although we did not observe a significant group-wide increase in IL-6 levels as a result of polytrauma, the presence of other injuries appears to have reduced the prognostic accuracy of this marker in identifying patients at risk for developing elevated ICP. In support of this possibility, we observed that patients with orthopedic injuries in the absence of TBI had significantly higher serum IL-6 levels than those observed in healthy volunteers. Extracranial sources of IL-6 such as those observed in the orthopedic injury patients may have contributed to the increase in IL-6 levels seen in polytrauma patients whose ICP remained below 20 mm Hg (Figure 3C), making them indistinguishable from patients whose ICP ≥ 25 mm Hg. However, due to the variety of extracranial injuries observed in the polytrauma patients (e.g. ruptured spleen, burns, fractures, etc.), it is not clear if different cut-off values, time of sampling, or further sub-classification based on type(s) of bodily injury, could be employed to increase the sensitivity of this marker for use in polytrauma.

Although IL-6 appears to lack sufficient specificity to diagnose or detect brain injury, our study suggests that it may have utility as a biomarker capable of predicting patients with isolated TBI who are at risk for developing elevated ICP, being able to identify these patients up to 2 days prior to the recorded increases in ICP. Previous studies have indicated that the CNS specific proteins glial fibrillary acidic protein (GFAP), cleaved tau (C-tau) and neuron-specific enolase (NSE) also have some prognostic capacity in identifying TBI patients at risk for developing elevated ICP [45-47]. For example, Zemlan et al., have reported that a high initial C-tau level in the CSF of TBI patients is a good predictor of elevated ICP [45]. However, recent studies have shown that the process of CSF drainage can not only change ICP, but depending on the method of drainage (continuous versus intermittent), can also dramatically influence CSF biomarker levels [48]. In the serum, S100B and GFAP concentrations have been shown to be lower in patients with ICP < 25 mm Hg than in patients with ICP > 25 mm Hg [46]. While S100B has been demonstrated to have extracranial sources, serum GFAP levels are not increased by non-head injuries [46,49]. In addition to these CNS proteins, we have recently demonstrated that the levels of serum ceruloplasmin, the major copper carrier in the blood, transiently decrease in TBI patients who will develop elevated ICP, and that this reduction can be used as a sensitive prognostic marker of subsequent ICP increases [50]. It is therefore suggested that a combination of biomarkers could be developed that couples IL-6, other identified biomarkers, and routine diagnostic evaluation of patient injuries, to create a biomarker signature that predicts the occurrence of elevated ICP with both high sensitivity and specificity.

## Conclusions

Our results indicate that the initial level of serum IL-6 (within 17 hours of injury) can be used to identify isolated TBI patients at risk for developing elevated intracranial pressure. As this increase was observed, on average, 2 days prior to the recorded increase in ICP, this would allow sufficient time for physicians and caretakers to allocate resources and/or transport the patient so that appropriate ICP management procedures can be employed.

## List of abbreviations

AIS: abbreviated injury scale; c-tau: cleaved tau; GCS: Glasgow coma scale; GFAP: glial fibrillary acidic protein; ELISA: enzyme-linked immunosorbent assay; ICP: intracranial pressure; TBI: traumatic brain injury.

## Competing interests

The authors declare that they have no competing interests.

## Authors' contributions

GWH *c*ontributed to the overall design of the study and assisted in the interpretation of the resultant data, was responsible for maintaining the standards and upholding the reporting requirements for the use of human subjects, drafted and revised the article. ANM contributed to the acquisition and analysis of all data presented in the manuscript, prepared the presentation materials and performed the statistical analyses, drafted and revised the article. JPM contributed to the design of the Luminex experimentation and assisted in the analysis of the resultant data, and revised the article. LS contributed to the acquisition and analysis of the Luminex data. NHW contributed to the acquisition of the serum samples and analysis of the patient data used in this study. GLC made substantial contributions to the conception and overall design of the study, and revised the article. PKD made substantial contributions to the conception and overall design of the study, in the interpretation of the resultant data, drafted and revised the article. All authors have read and approved the final version of the manuscript.

## References

[B1] Stocchetti N Colombo A Ortolano F Videtta W Marchesi R Longhi L Time course of intracranial hypertension after traumatic brain injury J Neurotrauma 2007 24 1339 1346 10.1089/neu.2007.0300 17711395

[B2] Dearden NM Mechanisms and prevention of secondary brain damage during intensive care Clin Neuropathol 1998 17 221 228 9707338

[B3] Jantzen JP Prevention and treatment of intracranial hypertension Best Pract Res Clin Anaesthesiol 2007 21 517 538 10.1016/j.bpa.2007.09.001 18286835

[B4] Kadhim HJ Duchateau J Sebire G Cytokines and brain injury: invited review J Intensive Care Med 2008 23 236 249 10.1177/0885066608318458 18504260

[B5] Libby P Inflammation in atherosclerosis Nature 2002 420 868 874 10.1038/nature01323 12490960

[B6] Yamagami H Kitagawa K Nagai Y Hougaku H Sakaguchi M Kuwabara K Higher levels of interleukin-6 are associated with lower echogenicity of carotid artery plaques Stroke 2004 35 677 681 10.1161/01.STR.0000116876.96334.82 14752126

[B7] Patel RT Deen KI Youngs D Warwick J Keighley MR Interleukin 6 is a prognostic indicator of outcome in severe intra-abdominal sepsis Br J Surg 1994 81 1306 1308 10.1002/bjs.1800810914 7953393

[B8] Minambres E Cemborain A Sanchez-Velasco P Gandarillas M az-Reganon G Sanchez-Gonzalez U Correlation between transcranial interleukin-6 gradient and outcome in patients with acute brain injury Crit Care Med 2003 31 933 938 10.1097/01.CCM.0000055370.66389.59 12627008

[B9] Berger RP Ta'asan S Rand A Lokshin A Kochanek P Multiplex assessment of serum biomarker concentrations in well-appearing children with inflicted traumatic brain injury Pediatr Res 2008 65 97 102 10.1203/PDR.0b013e31818c7e27 18787505

[B10] Knaus WA Draper EA Wagner DP Zimmerman JE APACHE II: a severity of disease classification system Crit Care Med 1985 13 818 829 10.1097/00003246-198510000-00009 3928249

[B11] Teasdale G Jennett B Assessment of coma and impaired consciousness. A practical scale Lancet 1974 2 81 84 10.1016/S0140-6736(74)91639-0 4136544

[B12] Association for the Advancement of Automotive Medicine Abbreviated Injury Scale (AIS) 2005 - Update 2008 2008 Barrington, IL: AAAM Publications PMC3256842 22105401

[B13] Bell MJ Kochanek PM Doughty LA Carcillo JA Adelson PD Clark RS Comparison of the interleukin-6 and interleukin-10 response in children after severe traumatic brain injury or septic shock Acta Neurochir Suppl (Wien) 1997 70 96 97 10.1007/978-3-7091-6837-0_30 9416290

[B14] Kossmann T Hans VH Imhof HG Stocker R Grob P Trentz O Intrathecal and serum interleukin-6 and the acute-phase response in patients with severe traumatic brain injuries Shock 1995 4 311 317 10.1097/00024382-199511000-00001 8595516

[B15] Cui X Kalsotra A Robida AM Matzilevich D Moore AN Boehme CL Expression of cytochromes P450 4F4 and 4F5 in infection and injury models of inflammation Biochim Biophys Acta 2003 1619 325 331 12573492 10.1016/s0304-4165(02)00491-9

[B16] Basu A Krady JK O'Malley M Styren SD DeKosky ST Levison SW The type 1 interleukin-1 receptor is essential for the efficient activation of microglia and the induction of multiple proinflammatory mediators in response to brain injury J Neurosci 2002 22 6071 6082 12122068 10.1523/JNEUROSCI.22-14-06071.2002 PMC6757935

[B17] John GR Lee SC Song X Rivieccio M Brosnan CF IL-1-regulated responses in astrocytes: relevance to injury and recovery Glia 2005 49 161 176 10.1002/glia.20109 15472994

[B18] Murphy PG Grondin J Altares M Richardson PM Induction of interleukin-6 in axotomized sensory neurons J Neurosci 1995 15 5130 5138 7623140 10.1523/JNEUROSCI.15-07-05130.1995 PMC6577897

[B19] Ringheim GE Burgher KL Heroux JA Interleukin-6 mRNA expression by cortical neurons in culture: evidence for neuronal sources of interleukin-6 production in the brain J Neuroimmunol 1995 63 113 123 10.1016/0165-5728(95)00134-4 8550808

[B20] Goodman JC Van M Gopinath SP Robertson CS Pro-inflammatory and pro-apoptotic elements of the neuroinflammatory response are activated in traumatic brain injury Acta Neurochir Suppl 2008 102 437 439 full_text 19388362 10.1007/978-3-211-85578-2_85

[B21] Csuka E Morganti-Kossmann MC Lenzlinger PM Joller H Trentz O Kossmann T IL-10 levels in cerebrospinal fluid and serum of patients with severe traumatic brain injury: relationship to IL-6, TNF-alpha, TGF-beta1 and blood-brain barrier function J Neuroimmunol 1999 101 211 221 10.1016/S0165-5728(99)00148-4 10580806

[B22] Kossmann T Hans VH Imhof HG Stocker R Grob P Trentz O Intrathecal and serum interleukin-6 and the acute-phase response in patients with severe traumatic brain injuries Shock 1995 4 311 317 10.1097/00024382-199511000-00001 8595516

[B23] Hans VH Kossmann T Joller H Otto V Morganti-Kossmann MC Interleukin-6 and its soluble receptor in serum and cerebrospinal fluid after cerebral trauma Neuroreport 1999 10 409 412 10203344 10.1097/00001756-199902050-00036

[B24] Singhal A Baker AJ Hare GM Reinders FX Schlichter LC Moulton RJ Association between cerebrospinal fluid interleukin-6 concentrations and outcome after severe human traumatic brain injury J Neurotrauma 2002 19 929 937 10.1089/089771502320317087 12225653

[B25] Chiaretti A Genovese O Aloe L Antonelli A Piastra M Polidori G Interleukin 1beta and interleukin 6 relationship with paediatric head trauma severity and outcome Childs Nerv Syst 2005 21 185 193 10.1007/s00381-004-1032-1 15455248

[B26] Winter CD Pringle AK Clough GF Church MK Raised parenchymal interleukin-6 levels correlate with improved outcome after traumatic brain injury Brain 2004 127 315 320 10.1093/brain/awh039 14645145

[B27] Chiaretti A Antonelli A Mastrangelo A Pezzotti P Tortorolo L Tosi F Interleukin-6 and nerve growth factor upregulation correlates with improved outcome in children with severe traumatic brain injury J Neurotrauma 2008 25 225 234 10.1089/neu.2007.0405 18352836

[B28] Arand M Melzner H Kinzl L Bruckner UB Gebhard F Early inflammatory mediator response following isolated traumatic brain injury and other major trauma in humans Langenbecks Arch Surg 2001 386 241 248 10.1007/s004230100204 11466564

[B29] Harris TB Ferrucci L Tracy RP Corti MC Wacholder S Ettinger WH Jr Associations of elevated interleukin-6 and C-reactive protein levels with mortality in the elderly Am J Med 1999 106 506 512 10.1016/S0002-9343(99)00066-2 10335721

[B30] Oto J Suzue A Inui D Fukuta Y Hosotsubo K Torii M Plasma proinflammatory and anti-inflammatory cytokine and catecholamine concentrations as predictors of neurological outcome in acute stroke patients J Anesth 2008 22 207 212 10.1007/s00540-008-0639-x 18685925

[B31] Stangl M Zerkaulen T Theodorakis J Illner W Schneeberger H Land W Influence of brain death on cytokine release in organ donors and renal transplants Transplant Proc 2001 33 1284 1285 10.1016/S0041-1345(00)02479-9 11267293

[B32] Sullivan NJ Sasser AK Axel AE Vesuna F Raman V Ramirez N Interleukin-6 induces an epithelial-mesenchymal transition phenotype in human breast cancer cells Oncogene 2009 28 2940 2947 10.1038/onc.2009.180 19581928 PMC5576031

[B33] Fernandez-Real JM Vayreda M Richart C Gutierrez C Broch M Vendrell J Circulating interleukin 6 levels, blood pressure, and insulin sensitivity in apparently healthy men and women J Clin Endocrinol Metab 2001 86 1154 1159 10.1210/jc.86.3.1154 11238501

[B34] Wetmore JB Lovett DH Hung AM Cook-Wiens G Mahnken JD Sen S Associations of interleukin-6, C-reactive protein and serum amyloid A with mortality in haemodialysis patients Nephrology (Carlton) 2008 13 593 600 10.1111/j.1440-1797.2008.01021.x 18826487 PMC3375899

[B35] Ali C Nicole O Docagne F Lesne S MacKenzie ET Nouvelot A Ischemia-induced interleukin-6 as a potential endogenous neuroprotective cytokine against NMDA receptor-mediated excitotoxicity in the brain J Cereb Blood Flow Metab 2000 20 956 966 10.1097/00004647-200006000-00008 10894179

[B36] Carlson NG Wieggel WA Chen J Bacchi A Rogers SW Gahring LC Inflammatory cytokines IL-1 alpha, IL-1 beta, IL-6, and TNF-alpha impart neuroprotection to an excitotoxin through distinct pathways J Immunol 1999 163 3963 3968 10490998

[B37] Penkowa M Giralt M Carrasco J Hadberg H Hidalgo J Impaired inflammatory response and increased oxidative stress and neurodegeneration after brain injury in interleukin-6-deficient mice Glia 2000 32 271 285 10.1002/1098-1136(200012)32:3<271::AID-GLIA70>3.0.CO;2-5 11102968

[B38] Zweig MH Campbell G Receiver-operating characteristic (ROC) plots: a fundamental evaluation tool in clinical medicine Clin Chem 1993 39 561 577 8472349

[B39] Cavaillon JM Cytokines and macrophages Biomed Pharmacother 1994 48 445 453 10.1016/0753-3322(94)90005-1 7858154

[B40] Beagley KW Elson CO Cells and cytokines in mucosal immunity and inflammation Gastroenterol Clin North Am 1992 21 347 366 1512047

[B41] Zhang Y Lin JX Vilcek J Synthesis of interleukin 6 (interferon-beta 2/B cell stimulatory factor 2) in human fibroblasts is triggered by an increase in intracellular cyclic AMP J Biol Chem 1988 263 6177 6182 2452159

[B42] Yoshizaki K Nishimoto N Matsumoto K Tagoh H Taga T Deguchi Y Interleukin 6 and expression of its receptor on epidermal keratinocytes Cytokine 1990 2 381 387 10.1016/1043-4666(90)90069-6 2129417

[B43] Loppnow H Libby P Comparative analysis of cytokine induction in human vascular endothelial and smooth muscle cells Lymphokine Res 1989 8 293 299 2789322

[B44] Hensler T Sauerland S Bouillon B Raum M Rixen D Helling HJ Association between injury pattern of patients with multiple injuries and circulating levels of soluble tumor necrosis factor receptors, interleukin-6 and interleukin-10, and polymorphonuclear neutrophil elastase J Trauma 2002 52 962 970 10.1097/00005373-200205000-00023 11988666

[B45] Zemlan FP Jauch EC Mulchahey JJ Gabbita SP Rosenberg WS Speciale SG C-tau biomarker of neuronal damage in severe brain injured patients: association with elevated intracranial pressure and clinical outcome Brain Res 2002 947 131 139 10.1016/S0006-8993(02)02920-7 12144861

[B46] Pelinka LE Kroepfl A Leixnering M Buchinger W Raabe A Redl H GFAP versus S100B in serum after traumatic brain injury: relationship to brain damage and outcome J Neurotrauma 2004 21 1553 1561 10.1089/neu.2004.21.1553 15684648

[B47] Herrmann M Jost S Kutz S Ebert AD Kratz T Wunderlich MT Temporal profile of release of neurobiochemical markers of brain damage after traumatic brain injury is associated with intracranial pathology as demonstrated in cranial computerized tomography J Neurotrauma 2000 17 113 122 10.1089/neu.2000.17.113 10709869

[B48] Shore PM Thomas NJ Clark RS Adelson PD Wisniewski SR Janesko KL Continuous versus intermittent cerebrospinal fluid drainage after severe traumatic brain injury in children: effect on biochemical markers J Neurotrauma 2004 21 1113 1122 10.1089/neu.2004.21.1113 15453982

[B49] Dash PK Zhao J Hergenroeder GW Moore AN Biomarkers fof the diagnosis, prognosis, and evaluation of treatment efficacy for traumatic brain injury Neurotherapeutics 7 100 114 10.1016/j.nurt.2009.10.019 20129502 PMC5084117

[B50] Dash PK Redell JB Hergenroeder GW Zhao J Clifton GL Moore AN Serum ceruloplasmin and copper are early biomarkers of elevated intracranial pressure J Neurosci Res in press 10.1002/jnr.22336 20091772

